# Estrogen modulates vascular smooth muscle cell function through downregulation of SIRT1

**DOI:** 10.18632/oncotarget.22546

**Published:** 2017-11-10

**Authors:** Chien-Hsing Lee, Sheng-Chiang Su, Chi-Fu Chiang, Chu-Yen Chien, Chia-Chen Hsu, Tzu-Yi Yu, Shih-Ming Huang, Yi-Shing Shieh, Hong-Wei Kao, Chien-Sung Tsai, Yi-Jen Hung, Chih-Yuan Lin

**Affiliations:** ^1^ Division of Endocrinology and Metabolism, Department of Internal Medicine, Tri-Service General Hospital, National Defense Medical Center, Taipei, Taiwan; ^2^ Graduate Institute of Medical Sciences, National Defense Medical Center, Taipei, Taiwan; ^3^ Department of Biochemistry, National Defense Medical Center, Taipei, Taiwan; ^4^ Department of Oral Diagnosis and Pathology, Tri-Service General Hospital, National Defense Medical Center, Taipei, Taiwan; ^5^ Department of Pathology, Tri-Service General Hospital, National Defense Medical Center, Taipei, Taiwan; ^6^ Division of Cardiovascular Surgery, Department of Surgery, Tri-Service General Hospital, National Defense Medical Center, Taipei, Taiwan

**Keywords:** estrogen, sirtuin 1 (SIRT1), vascular smooth muscle cell, ovariectomy

## Abstract

**Background:**

There are sex differences in the incidence and severity of cardiovascular disease. Although an estrogen-mediated vasculoprotective effect is widely accepted, clinical trial results have been conflicting and the detailed mechanisms are still unclear. Sirtuin 1 (SIRT1), a class III histone deacetylase, may protect against vascular aging and atherosclerosis; however, the effects of estrogen on SIRT1 expression and vascular smooth muscle cell (VSMC) behavior remain unknown.

**Materials and Methods:**

We ovariectomized (OVX) female, wild-type, C57BL/6J mice, which were randomized into non-estrogen- and estrogen-supplemented groups. We also treated A7r5 VSMCs with 17-β-estradiol and resveratrol, a SIRT1 activator, *in vitro*, and measured the expression of SIRT1 and apoptotic markers, as well as proliferation, viability, and migration.

**Results:**

Aortic tissue from OVX mice exhibited marked VSMC hyperplasia and upregulation of SIRT1, which was reversed by 17-β-estradiol supplementation, as assessed by western blotting and immunohistochemical staining. *In vitro*, 17-β-estradiol downregulated SIRT1 expression in a dose- and time-dependent manner, increased apoptosis, and reduced proliferation, viability, and migration. Resveratrol reversed these effects through the activation of SIRT1. Estrogen appeared to mediate its effects through the Akt and ERK pathways.

**Conclusions:**

Estrogen may regulate cardiovascular health via the expression of SIRT1, possibly through the AKT and ERK signaling pathways.

## INTRODUCTION

Women generally have a lower risk for developing cardiovascular disease (CVD) than men of a similar age [1], but this protection is lost during menopause [2]. It is widely believed that estrogen is responsible for the protection of premenopausal women from CVD. However, hormone replacement therapy has failed to decrease CVD events in clinical studies, and conflicting results from clinical and experimental studies point to a complex relationship between vascular biology and estrogen hormones [3, 4]. From a recent analysis including 19 studies and 40,410 participants, oral estrogen therapy in postmenopausal population has no strong evidence in a primary or secondary prevention benefits associated with lower rates of all-cause mortality or cardiovascular outcomes. On the contrary, oral estrogen therapy is actually associated with higher rates of venous thromboembolism and stroke in primary prevention populations [5]. The decline of estrogen is a natural course of aging women, but estrogen supplement did not show significant benefits in this population. Thus, the cardiovascular actions of estrogen are complex and incompletely understood.

Sirtuins are a highly conserved family of histone/protein deacetylases that can prolong the lifespans of organisms [6, 7]. Sirtuin 1 (SIRT1) garnered extensive attention as a mediator of health and longevity in response to calorie restriction [8]. Recent studies have found that SIRT1 plays a protective role in CVD through a variety of mechanisms, including reducing inflammation, improving endothelial function, delaying cellular senescence, and defending against oxidative stress [9–11]. Studies have also found that endothelial SIRT1 is an anti-atherosclerotic factor [12] and SIRT1 in vascular smooth muscle cells protectes against aortic stiffness [13]. In addition, SIRT1 modulates the generation of endothelial nitric oxide, a protective factor for endothelial cells that exerts an anti-atherosclerotic effect [14].

The profound protective actions of estrogen on the vasculature are multifaceted. Estrogen is likely to act directly on the endothelium and vascular smooth muscle, through both rapid signaling pathways and genomic mechanisms [15]. Most studies on the effects of estrogen on the vascular wall have focused on endothelial cells [16–18]. Vascular smooth muscle cells (VSMCs) play a dominant role in the functional and structural changes of the arterial walls in response to atherogenic factors, and SIRT1 is a critical protective protein in VSMCs, where it reduces DNA damage, apoptosis, and cell senescence [19]. However, the effects of estrogen on the regulation of SIRT1 in VSMCs remain unknown.

Current evidence indicates that older women should not take hormone therapy owing to its detrimental effects on cardiovascular system [20, 21]. The level of SIRT1 is decreased in both transcriptional and postranscriptional conditions during aging and is an important component of aging-related diseases [22]. In this study, we hypothesized that estrogen can modulates VSMCs function through downregulation of SIRT1. To verify the hypothesis, we compare the expression of SIRT1 in sham-operated and ovariectomized (OVX) mice, as well as OVX mice that received 17-β-estradiol (E2) supplementation *in vivo*, and to subsequently explore E2-mediated changes in cell behavior *in vitro*.

## RESULTS

### Marked VSMC proliferation and upregulation of SIRT1 in OVX mice

**Figure 1:**
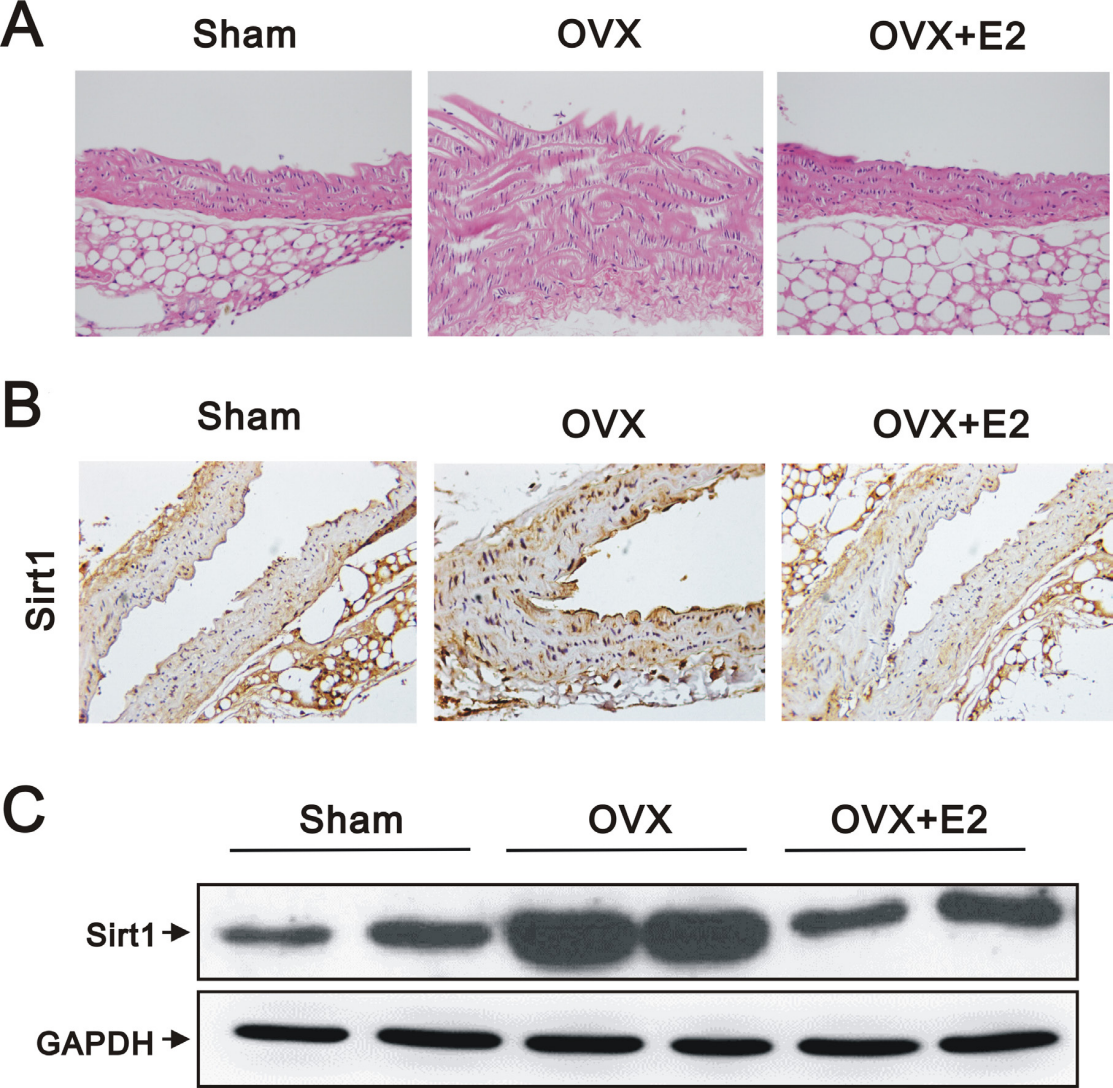
Histology, IHC, and western blot analyses of aortas from sham, OVX, and OVX+E2 mice (**A**) Representative images of hematoxylin and eosin stained, paraffin-embedded aortas (original magnification, 400×), (**B**) representative IHC staining for SIRT1 expression (original magnification, 400×), and (**C**) western blots using anti-SIRT1 and anti-GAPDH antibodies of aortic tissues from sham, OVX, and OVX+E2 mice.

VSMC proliferation and migration are crucial events in the pathophysiology of vascular diseases [23]. In OVX mice, the aortic tissue sections stained with hematoxylin and eosin exhibited increased wall thickness and vascular smooth muscle hyperplasia by subjective observation (Figure 1A). We also found increased expression of SIRT1 by immunohistochemical analysis (IHC) (Figure 1B) and western blotting (Figure 1C) in the aortic tissues of OVX, compared to sham-operated, mice. However, the effects of OVX were reversed by E2 administration (Figure 1A–1C).

### Estrogen downregulates SIRT1 expression in A7r5 cells

**Figure 2:**
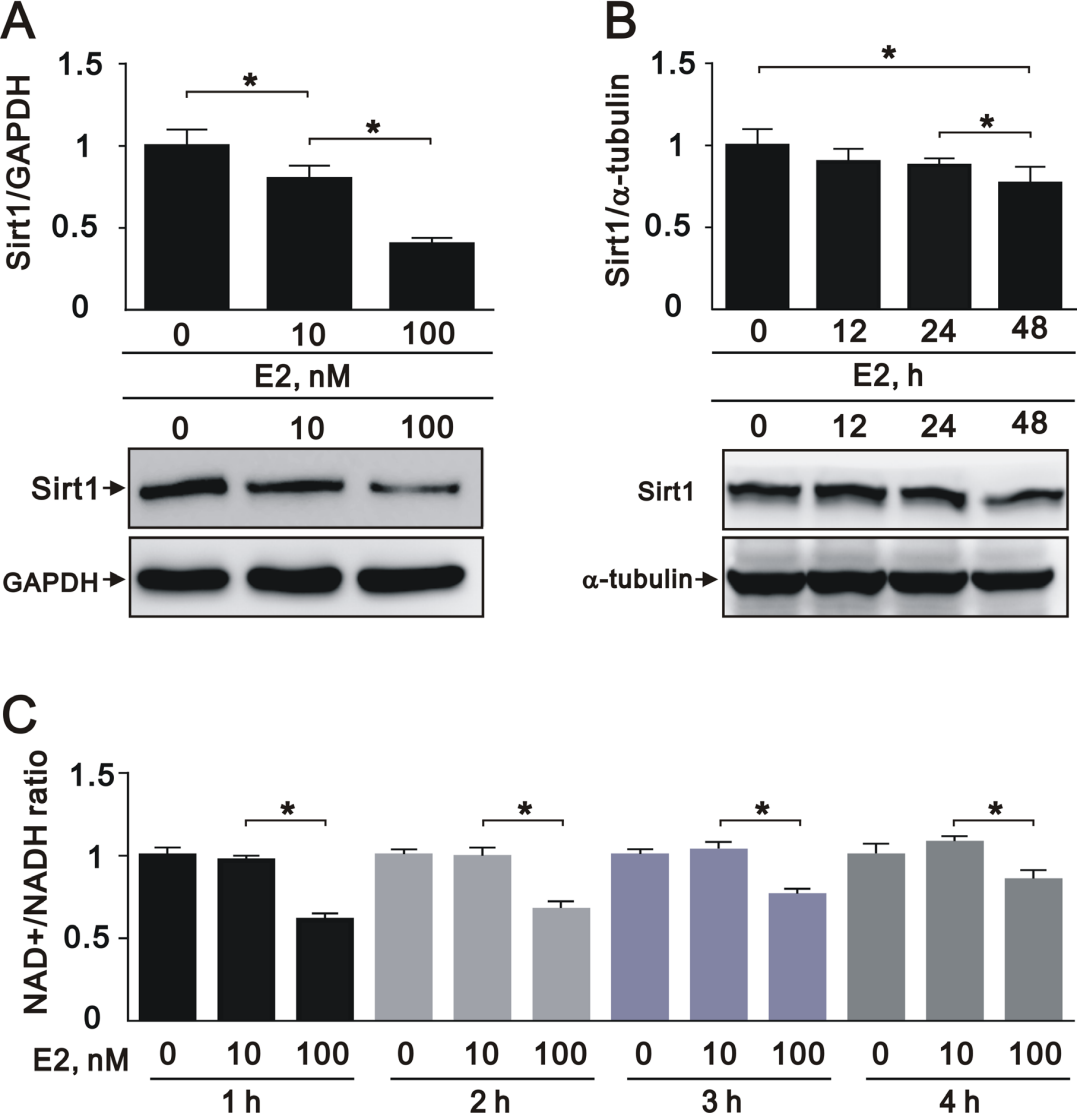
E2 treatment downregulated SIRT1 expression in the A7r5 rat smooth muscle cell line We treated A7r5 cells with 10 nM and 100 nM E2 (**A**), or over a time course with 10 nM E2 (0, 12, 24, and 48 h) (**B**); we subjected the samples to western blot analysis to quantify SIRT1, GAPDH, and α-tubulin protein expression. (**C**) We analyzed the NAD^+^/NADH ratio in A7r5 cells subjected to the indicated treatments. Data are expressed as mean ± standard deviation (SD). Representative data shown are from experiments performed independently at least 3 times. ^*^p < 0.05

We next found that E2 treatment decreased the expression of SIRT1 in a dose-dependent manner (10 nM and 100 nM) in A7r5 cells (Figure 2A). In addition, E2 treatment slightly decreased SIRT1 expression in a time-dependent manner (0, 12, 24, and 48 h) (Figure 2B). Given the role of SIRT1 as an NAD^+^-dependent protein deacetylase, we examined if the deacetylation capability of SIRT1 was affected by E2. In an NAD^+^/NADH assay, we found that E2 treatment (100 nM) significantly decreased the NAD^+^/NADH ratio (Figure 2C). These data suggested that E2 treatment downregulates SIRT1 expression in A7r5 cells.

The effects of estrogen on the apoptosis, proliferation, cell cycle status, and migration of A7r5 cells

**Figure 3:**
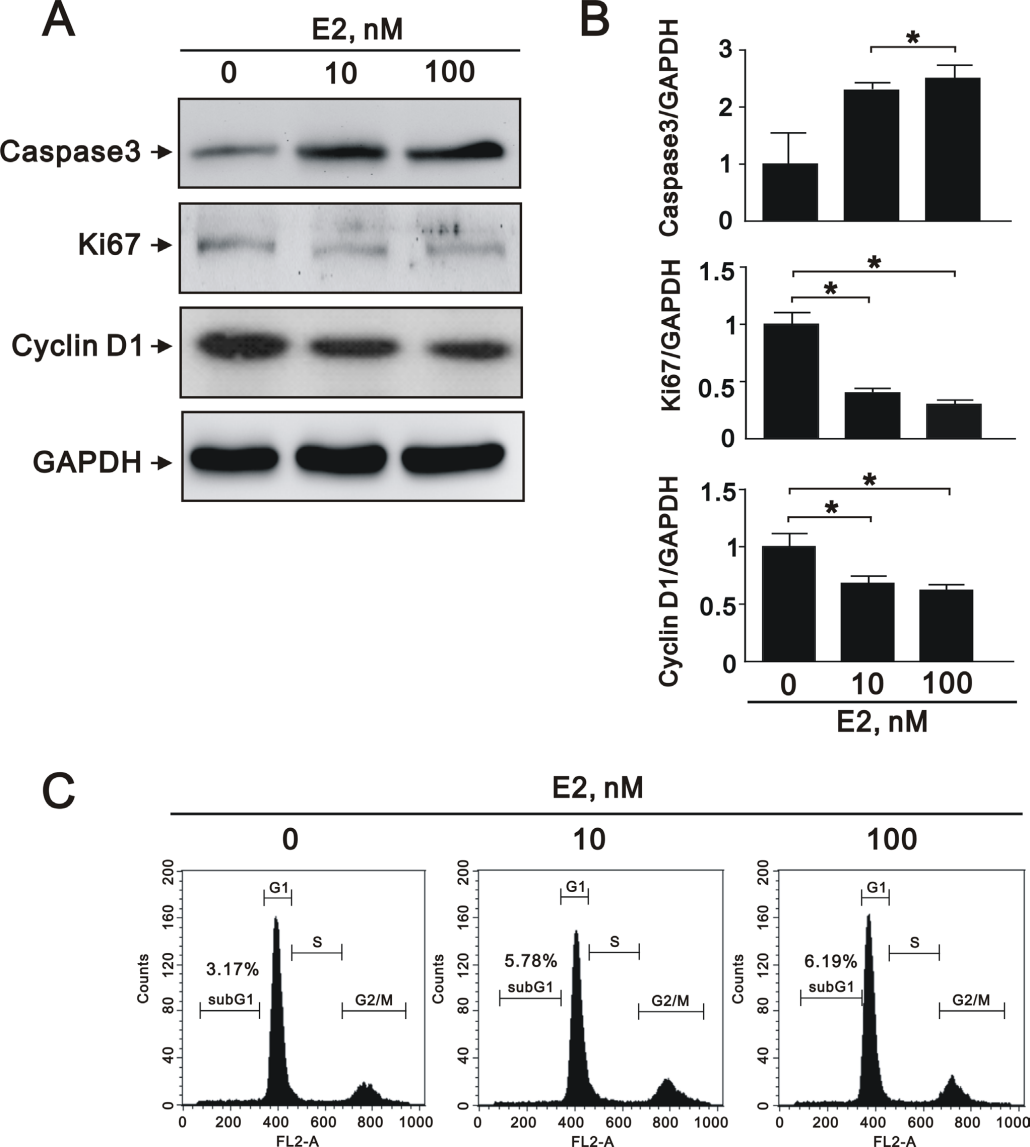
E2 treatment regulated A7r5 apoptosis, proliferation, and migration (**A**) Representative immunoblots of protein samples from A7r5 cells treated with 10 nM and 100 nM E2 that were stained with antibodies against caspase 3, Ki-67, cyclin D1, and GAPDH. (**B**) We quantified caspase 3, Ki-67, and cyclin D1 protein expression by computer-assisted densitometry analysis and presented their ratios to GAPDH, relative to the control group. (**C**) We performed flow cytometry on A7r5 cells treated with 10 nM and 100 nM E2, in order to assess the percentage in the G1 phase of the cell cycle.

**Figure 4:**
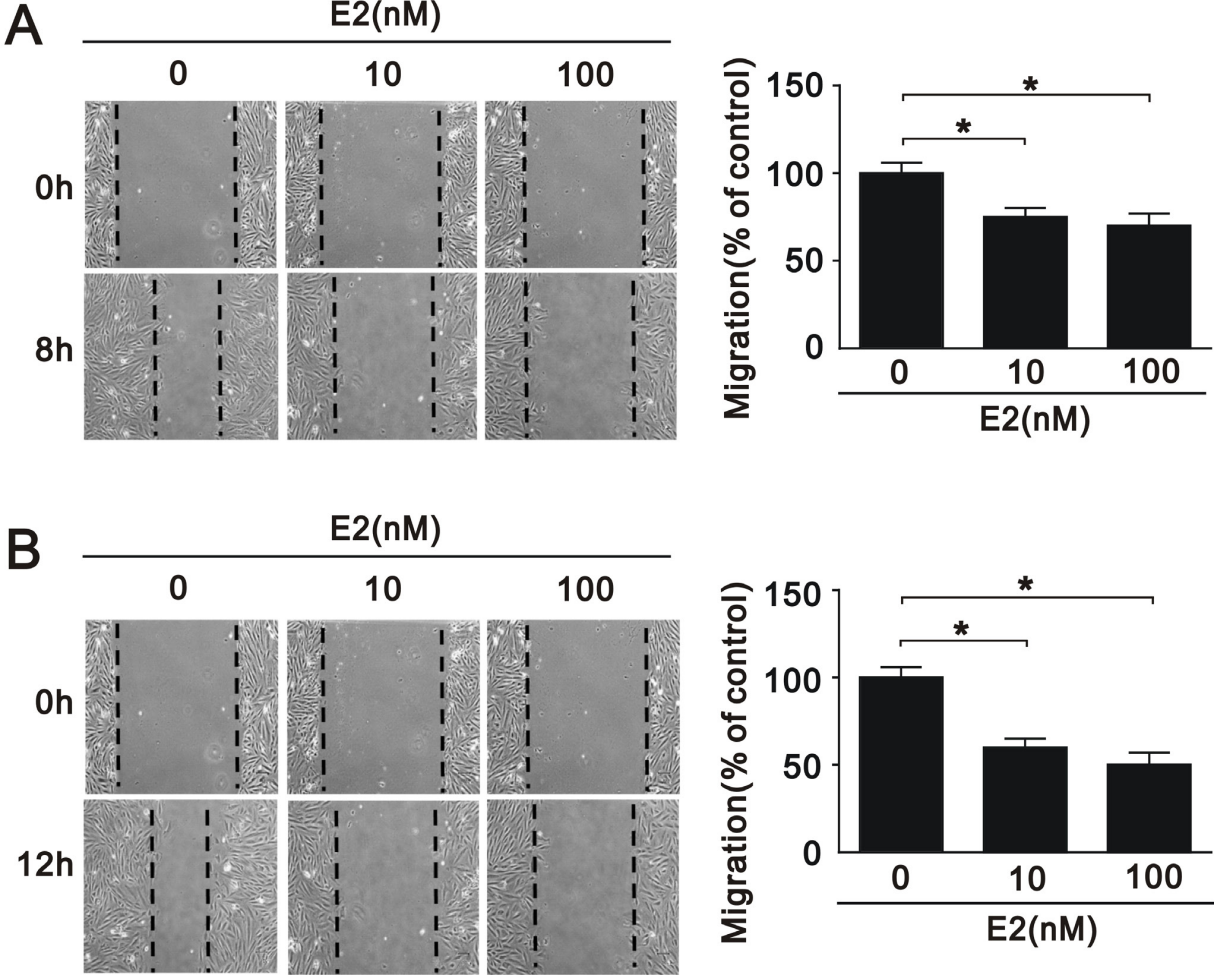
Migration of A7r5 cell treated with E2 Wound healing assays were performed on A7r5 cells treated with 10 nM and 100 nM E2 for (**A**) 8 h and (**B**) 12 h. Black arrows indicate the wound edge. The residual gap between the migrating cells from the opposing wound edge is expressed as a percentage of the initial scraped area. Data are expressed as mean ± SD. Representative data shown are from experiments performed independently at least 3 times. ^*^p < 0.05

VSMC apoptosis has been implicated in a number of deleterious consequences of atherosclerosis, including plaque rupture, vessel remodeling, coagulation, inflammation, and calcification [24]. To elucidate the effects of SIRT1 downregulation after E2 treatment in A7r5 cells, we performed cell apoptosis, proliferation, and migration assays. E2 treatment increased the expression of caspase 3, a marker of apoptosis, in a dose-dependent manner (10 nM and 100 nM) (Figure 3A and 3B), and decreased the expression of Ki-67, a marker of proliferation (Figure 3A and 3B). We also found that E2 treatment significantly decreased the expression of cyclin D1 (Figure 3A and 3B), an important regulator of cell cycle progression [25]. Subsequently, we examined the effect of E2 treatment on cell cycle regulation in A7r5 cells by flow cytometry; we found that E2 treatment increased cell cycle arrest in a dose-dependent manner (5.78% in 10 nM and 6.19% in 100 nM, respectively) (Figure 3C). Moreover, we found that E2 treatment decreased cell migration in a dose-dependent manner (10 nM and 100 nM) after 8 (Figure 4A) and 12 h (Figure 4B). Taken together, these results showed that E2 regulates cell apoptosis, proliferation, cell cycle progression, and migration.

### Treatment with a SIRT1 activator reverses the effects of estrogen in A7r5 cells

**Figure 5:**
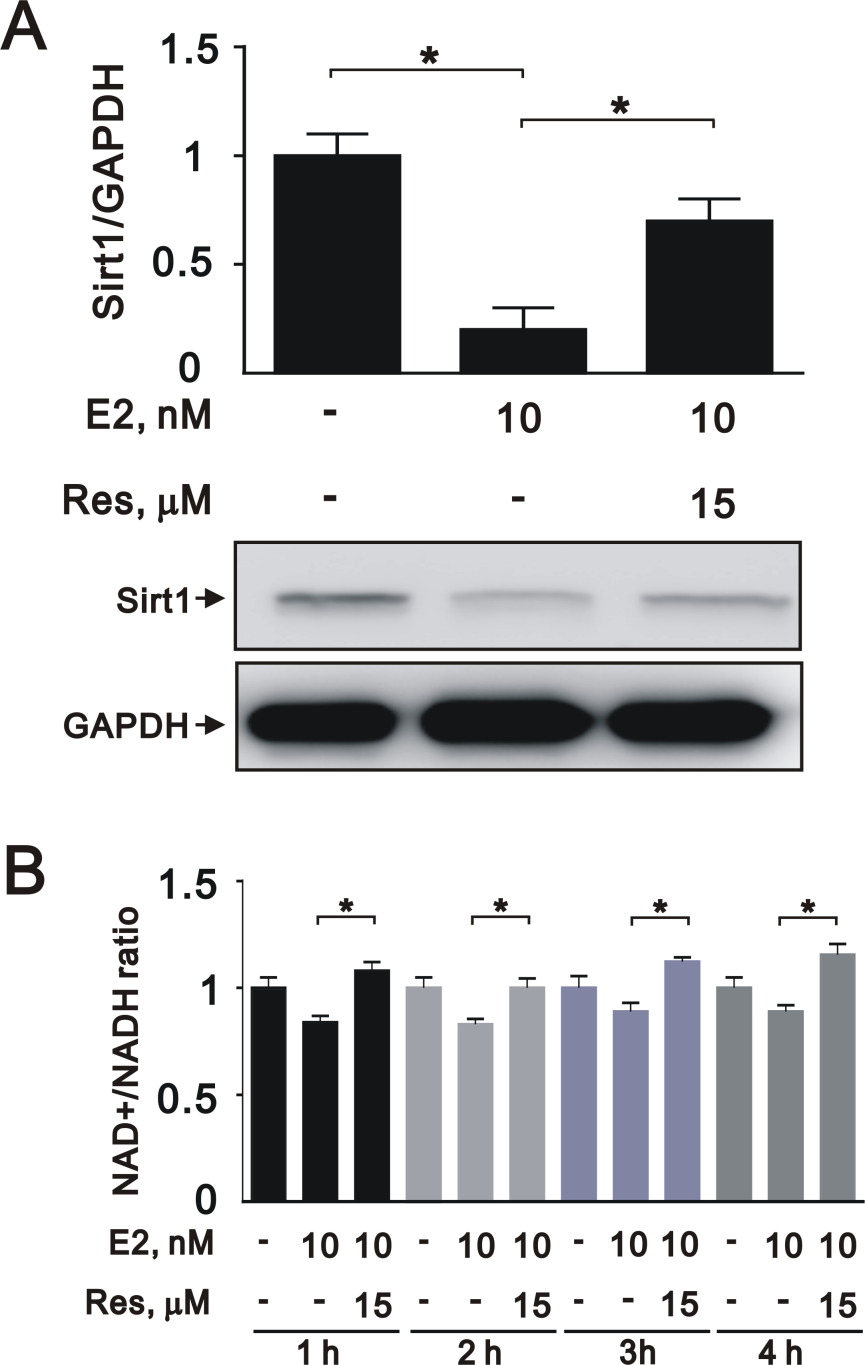
Resveratrol reversed the effects of E2 on SIRT1 expression in A7r5 cells (**A**) We treated A7r5 cells with 10 nM E2 with and without resveratrol (Res, 15 μM); we then performed western blot analyses to quantify the levels of SIRT1 and GAPDH. (**B**) We analyzed the NAD^+^/NADH ratio in the presence and absence of resveratrol. Data are expressed as mean ± SD. Representative data shown are from experiments performed independently at least 3 times. ^*^p < 0.05

**Figure 6:**
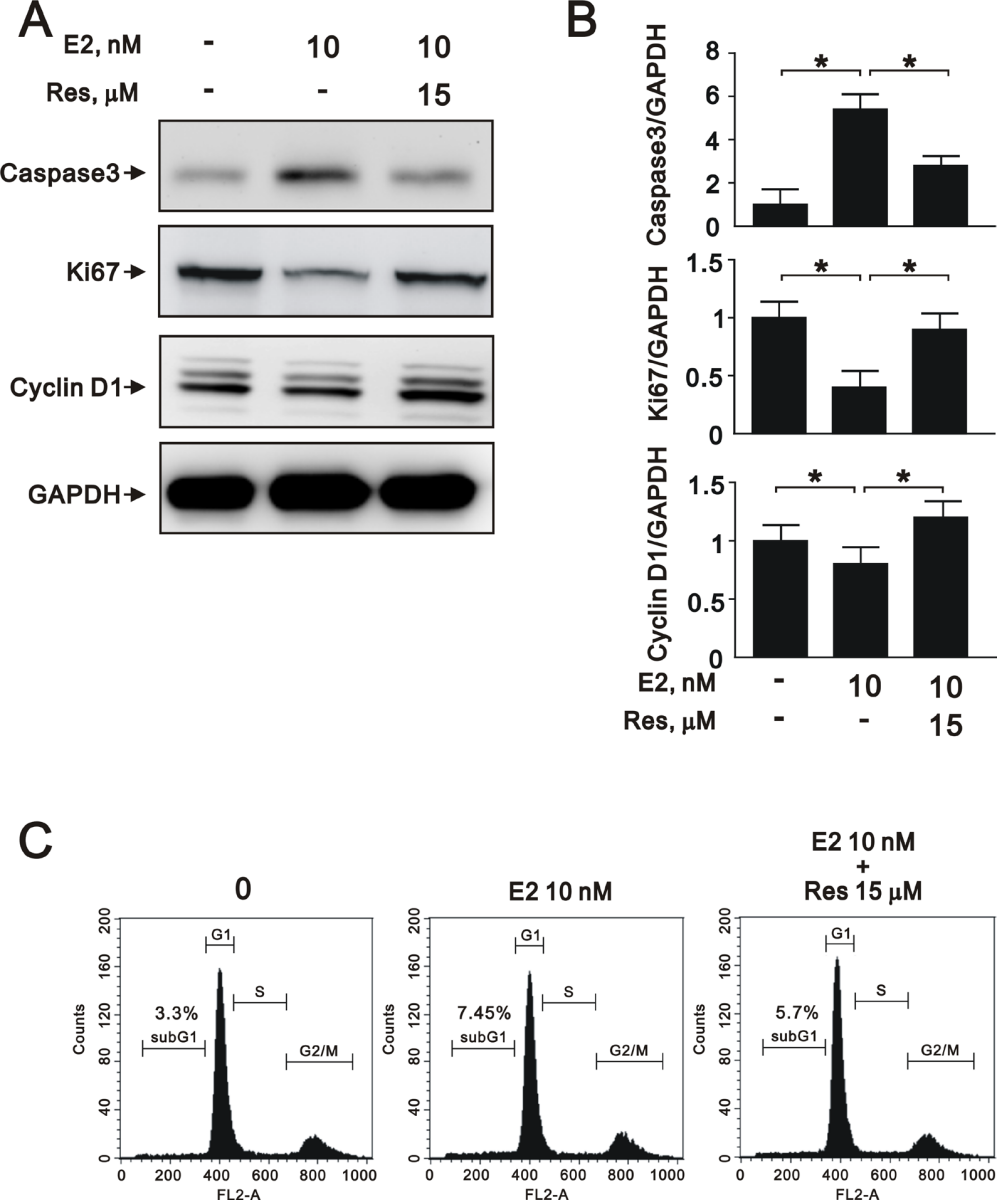
Resveratrol reversed E2-induced effects on proliferation, apoptosis, and cell cycle progression in A7r5 cells through SIRT1 activation (**A**) Representative immunoblots of protein samples from A7r5 cells exposed to 10 nM E2 with and without resveratrol (Res, 15 μM), labeled with antibodies against caspase 3, Ki-67, cyclin D1, and GAPDH. (**B**) We quantified caspase 3, Ki-67, and cyclin D1 protein expression by computer-assisted densitometry analysis and presented their ratios to GAPDH, relative to the control group. (**C**) Flow cytometric analysis of A7r5 cells in the G1 phase of the cell cycle after treatment with 10 nM E2 with and without resveratrol (Res, 15 μM).

**Figure 7:**
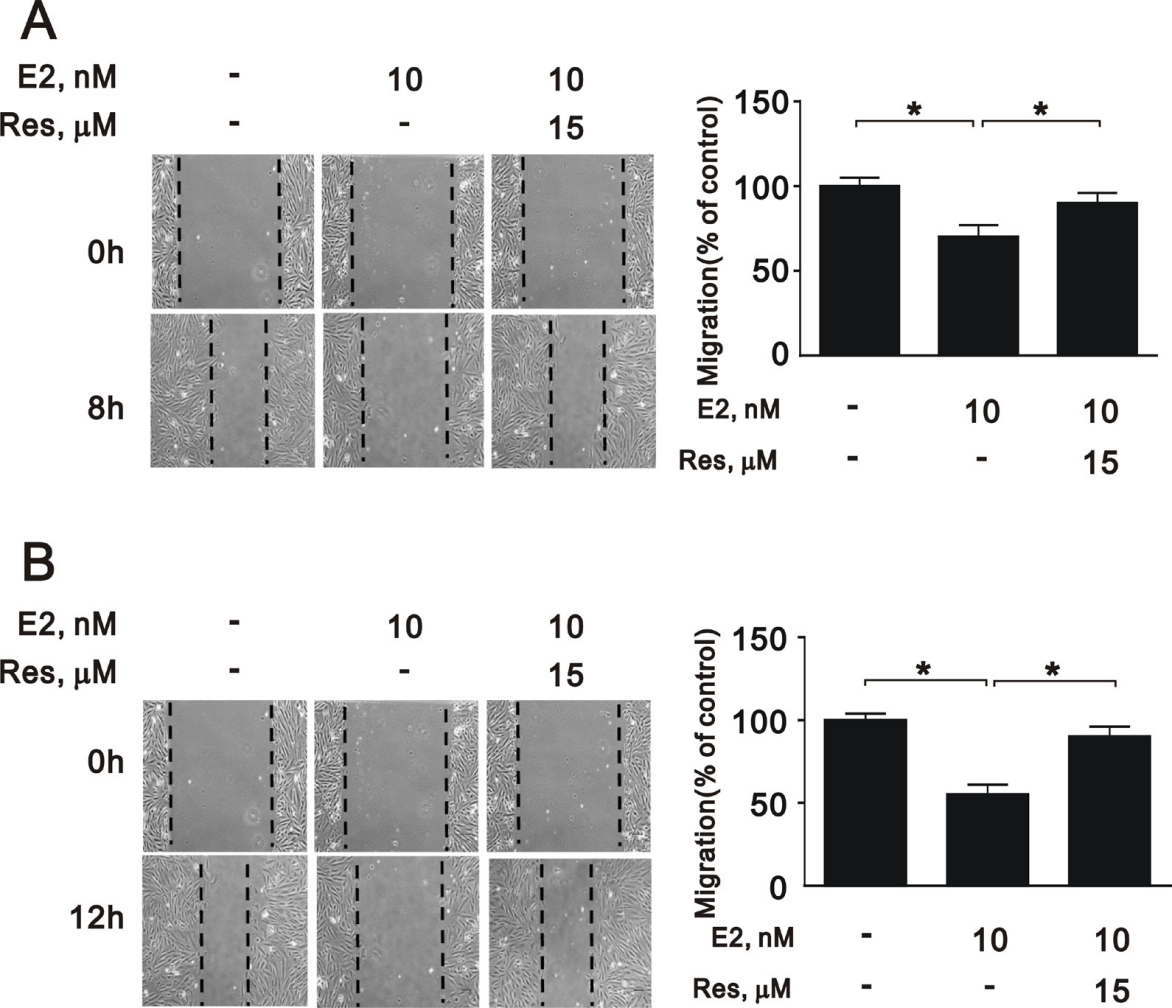
Migration of A7r5 cell treated with E2 in the presence and absence of resveratrol Wound healing assays were performed on A7r5 cells treated with 10 nM E2, with and without resveratrol (Res, 15 μM) after (**A**) 8 h and (**B**) 12 h. One representative experiment is shown. Black arrows indicate the wound edge. The residual gap between the migrating cells from the opposing wound edge is expressed as a percentage of the initial scraped area. Data are expressed as mean ± SD. Representative data shown are from experiments performed independently at least 3 times. ^*^p < 0.05

To further elucidate the role of SIRT1 after E2 treatment in A7r5 cells, we induced SIRT1 expression with resveratrol, a widely used SIRT1 activator. Treatment with resveratrol increased the expression of SIRT1 (Figure 5A) and the NAD^+^/NADH ratio (Figure 5B) that had been downregulated by E2 treatment. Moreover, resveratrol significantly reversed the effects of E2 on caspase 3, Ki-67, and cyclin D1 expression (Figure 6A and 6B) and cell cycle arrest (Figure 6C). We also found that resveratrol reversed the inhibition of cell migration caused by E2 treatment after 8 (Figure 7A) and 12 h (Figure 7B). Collectively, these data showed that E2 treatment regulates cell behavior through the regulation of SIRT1.

### Estrogen regulates SIRT1 expression and changes in cell behavior through the Akt and ERK pathways

**Figure 8:**
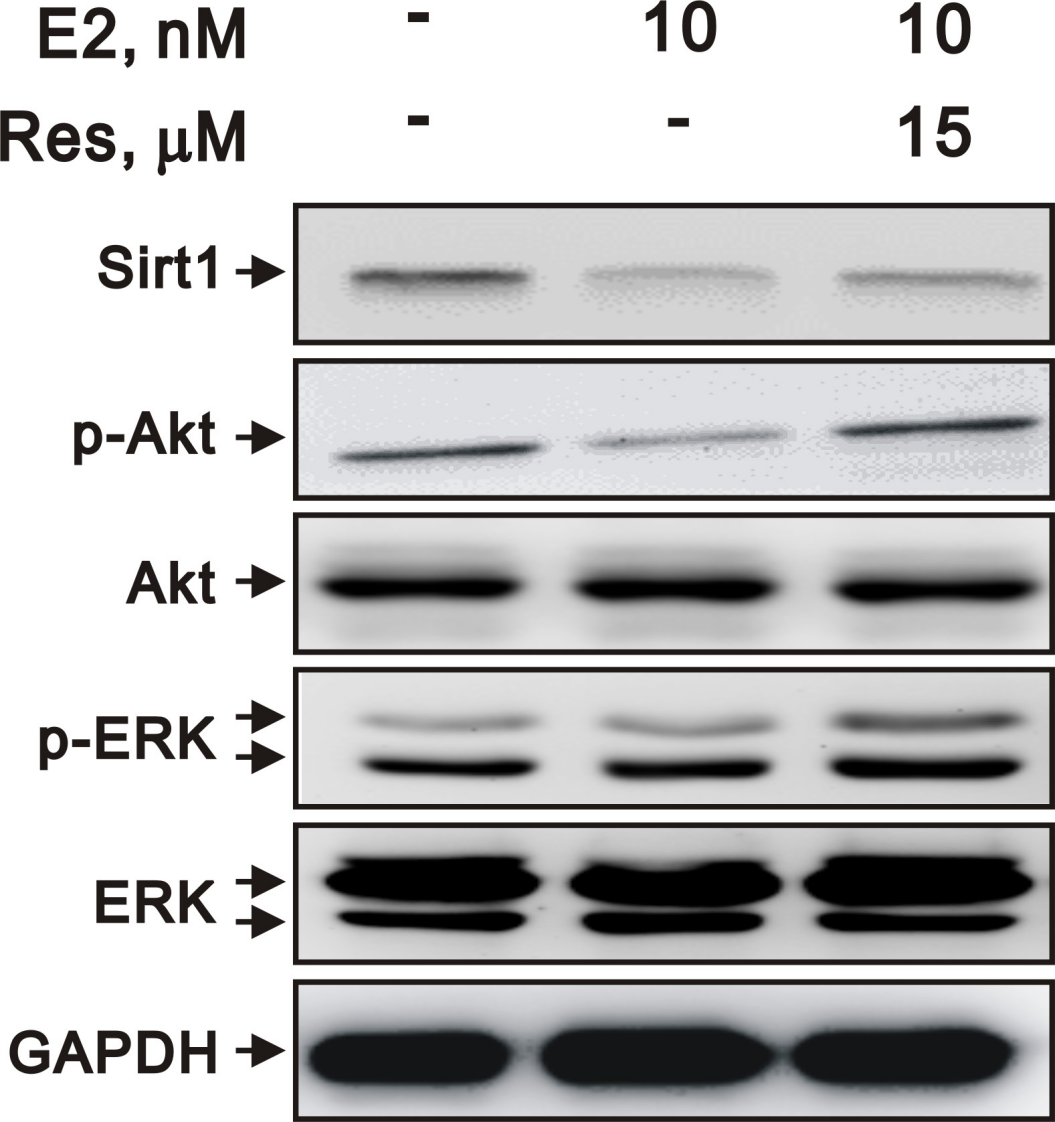
E2 and resveratrol regulated SIRT1 expression and downstream signaling Representative immunoblots of A7r5 cells treated with 10 nM E2, with and without resveratrol (Res, 15 μM), labeled with antibodies against SIRT1, p-Akt, Akt, p-ERK, ERK, and GAPDH.

To examine the mechanisms by which E2 inhibits VSMC proliferation, we assessed the phosphorylation of kinases that promote cell growth, including Akt and ERK. We found that E2 decreased the expression of SIRT1 and decreased the phosphorylation of Akt and ERK. However, resveratrol attenuated the downregulation of Akt and ERK phosphorylation (Figure 8). These data suggested that SIRT1 may be involved in the E2-mediated inhibition of VSMC proliferation through Akt and ERK signaling.

## DISCUSSION

In this study, we observed marked proliferation of aortic VSMCs, characterized by the upregulation of SIRT1, in OVX mice, which was reversed by E2 supplementation. *In vitro*, E2 downregulated the expression of SIRT1 in a dose- and time-dependent manner in A7r5 cells, and affected cell apoptosis, proliferation, viability, and migration. Resveratrol, the common-used SIRT1 activator [26], reversed these effects. Moreover, estrogen downregulated the expression of SIRT1 and could affect cell behavior through the Akt and ERK pathways. Our present work is valuable to show the effect of estrogen on VSMC through the modulation of SIRT1, which is considered as a vascular protective factor. More understanding about the basic mechanism of estrogen may provide evidence to create consensus and clarify strategies for post-menopausal hormone therapy.

The incidence and severity of cardiovascular disease vary between men and women and woman generally have a lower risk for developing CVD compared to men of similar age [1]. Across the industrialized world, women live 5–10 years longer than men because they develop CVD about 10 years later, possibly due to the protective effect of estrogen on the female circulatory system [2]. Epidemiological studies have shown that the onset of CVD, the major manifestation of atherosclerosis, occurs on an average 10 years later in premenopausal women than in men, with myocardial infarction occurring about 20 years later [27]. But this protection is lost during menopause, there is a 10-fold increase in CVD after menopause in women campared to only a 4.6-fold increase in the same age groups in men [27]. From the updated statistical data from AHA, the overall rate of death attributable to CVD was 222.9 per 100,000 Americans in 2013 with the death rates 269.8 for males and 184.8 for females [1].

Increases in the incidences of cardiovascular disease and metabolic syndrome in women following the onset of menopause have highlighted the role of estrogen as a cardiometabolic protective agent. Numerous animal studies have shown that estrogen protects against the development of atherosclerosis. Estrogen also inhibits the response to vascular injury in the mouse carotid injury model, by inhibiting the proliferation of VSMCs [28–30]. However, some large-scale clinical trials have demonstrated that estrogen may not always be beneficial for cardiovascular health and may, in some cases, be detrimental [21, 31–33]. Several current concepts including total hormone exposure time and underlying CAD risks, tissue specificity of sex hormones, the timing and the stage of atherosclerosis and the relationships with biomarkers and inflammation have been developed in the estrogen replacement therapy for menopausal women. All these issues are important and thus highlight the complexity of the effects of estrogen on the cardiovascular system [20]. Taken together, these findings underscore the complexity of the effects of estrogen on the cardiovascular system, including its ability to exert both potentially harmful and beneficial effects. Our present work is valuable to show the effect of estrogen on VSMC through the modulation of SIRT1, which is considered as a vascular protective factor. More understanding about the basic mechanism of estrogen may provide evidence to create consensus and clarify strategies for post-menopausal hormone therapy. The molecular mechanisms by which estrogen exerts its cardiovascular effects must be better understood before its clinical application.

SIRT1, a class III histone deacetylase, regulates multiple cellular functions, and may slow the development of atherosclerosis [34]. SIRT1 is a novel modulator of neointima formation due to arterial injuries; it decreases VSMC proliferation and migration through the downregulation of cyclin D1 and MMP-9 activity [23]. In human VSMCs, endogenous SIRT1 decreases with age, and loss of this protein directly contributes to the induction of cellular senescence and deficits in cellular functions, including impaired stress responses and reduced capacity for cell migration and proliferation [35]. Reduced SIRT1 expression is associated with increased apoptosis; VSMC apoptosis can increase atherosclerosis [36], with an increased necrotic core, reduced fibrous cap thickness, and foci of inflammation within the cap [37]. These previous reports are consistent with our *in vivo* and *in vitro* findings on the downregulation of SIRT1 by estrogen.

Both the genomic pathway and the rapid, non-nuclear pathway are responsible for the effects of estrogen on VSMCs [38–41]. The anti-atherogenic properties of estrogen have been shown to affect each component of the atherosclerotic cascade [42]. Estrogen also exerts an inhibitory effect on the growth and proliferation of VSMCs [43, 44], likely through inhibition of MAPK transactivation, nuclear transcription, and the expression of growth factors. E2 also causes rapid relaxation of endothelium-denuded vascular segments, suggesting direct effects on the VSMC contraction mechanism [45]. In particular, the direct effects of estrogen on VSMCs underline the importance of furthering our understanding of the molecular actions of estrogen. We previously found that a rapid, non-nuclear signaling pathway is required for the estrogen-mediated anti-proliferative and anti-migratory effects on VSMCs [38].

There are some limitations in this study. First, we used A7r5 cell line for *in vivo* study and to investigate the possible signaling pathway instead of primary culture of VSMCs. Second, we did not perform the *in vivo* and *in vitro* experiments in diseased or stressed model but in normal physiologic condition. Third, the concentration of E2 used in the *in vitro* study is higher than the usual physiological level, but it may minic high-dose estrogen supplement.

Ultimately, the decline in SIRT1 levels and functionality of the VSMCs treated with estrogen suggests a possible detrimental role for estrogen in vascular health. The protein expression of SIRT1 has been demonstrated to be highest in the embryo with progressive reductions associated with aging [46]. Moreover, the level of SIRT1 is decreased in both transcriptional and postranscriptional conditions during aging, accompanied by attenuated mitochondrial biogenesis, an important component of aging-related diseases [22]. During aging, the negative effects associated with natural decline may be exacerbated by the effects of estrogen. There are some controversies regarding the effects of estrogen on the regulation of SIRT1 in the vasculature [47, 48]. Our observations may partially explain the negative impact of estrogen replacement therapy on cardiovascular disease in some large-scale clinical trials. Improved understanding of the molecular actions of estrogen is required to optimize the development of hormonal treatments for postmenopausal women. In addition, the dose of the exogenous hormone may also be relevant.

## MATERIALS AND METHODS

### Animal model

Animals were bred and housed in accordance with the guidelines of the National Defense Medical Center of the Laboratory Animal Center, Taiwan, ROC. The animal experiments were approved by the local animal care committee of the National Defense Medical Center. After acclimatization, the female, wild-type, C57BL/6J mice (5–7 weeks of age) were randomly allocated into 3 groups: sham-operated, OVX, and OVX+E2. All of the animals were anesthetized with chloral hydrate (Sigma-Aldrich, St. Louis, MO, USA), and the OVX groups underwent bilateral ovariectomy via the dorsal route. The sham-operated control group was subjected to anesthesia and incision without ovariectomy. Starting one week later, OVX+E2 mice received intraperitoneal injection of 1.75 μg E2/25 g of body weight (Sigma-Aldrich), 3 times per week. Eighteen weeks later, we euthanized the mice and collected the aortic tissues for further analysis.

### IHC

We deparaffinized and rehydrated 4-μm sections of formalin-fixed, paraffin-embedded tissues, which were subjected to antigen retrieval. The tissue sections were incubated with hydrogen peroxide for 10 min at room temperature to quench endogenous peroxidases. After they were blocked in normal goat serum, the tissue sections were incubated at 4°C overnight with a primary anti-SIRT1 antibody (1:100 dilution). The slides were then washed with 0.5% Triton™ X-100 in phosphate-buffered saline (PBS) and incubated with horseradish peroxidase-conjugated secondary antibody for 1 h (Leica Biosystems, USA). Nuclei were counterstained with hematoxylin. As a negative control, we substituted normal goat serum for the primary antibody. For each section examined, we counted cells in 5 randomly selected fields.

### Western blot analysis

After various treatments, A7r5 cells and tissue lysates were prepared in lysis buffer containing 20 mM Tris-HCl (pH 7.5), 150 mM NaCl, 1 mM EDTA, 1 mM EGTA, 1% Triton™ X-100, 50 mM dithiothreitol, and complete protease inhibitor cocktail. The protein concentrations were assayed using a BCA protein assay kit. Equal amounts of total protein (30 μg) were loaded onto either 8% or 10% sodium dodecyl sulfate (SDS) polyacrylamide gels, and transferred to nitrocellulose membranes. After they were blocked with 5% nonfat milk in buffer containing 20 mM Tris-HCl, 150 mM NaCl (pH 7.5), and 0.1% TWEEN^®^ 20 (TBST), the blots were incubated with anti-SIRT1 (1:1,000), anti-Ki67 (1:3,000), anti-cyclin D1 (1:500), anti-caspase 3 (1:1,000), anti-p-AMPK (1:1,000), anti-AMPK (1:1,000), anti-p-ERK (1:1,000), anti-ERK (1:1,000), anti-p-AKT (1:1,000), anti-AKT (1:1,000), anti-ERα (1:1,000), or anti-GAPDH (1:10,000) antibody overnight at 4°C. After they were washed with TBST, the blots were incubated with the horseradish peroxidase-conjugated secondary antibody (1:5,000) for 1 h at room temperature. Immunoreactive bands were quantified using an enhanced chemiluminescence system of detection.

NAD+/NADH ratio determination

Sirtuins are a well-known, highly conserved, family of NAD^+^-dependent deacetylase that are involved in the regulation of life span from yeast to humans [49, 50]. Therefore, we investigated the NAD+/NADH ration to verify the effect or SIRT1 using a Biovision NAD^+^ and NADH quantification kit according to the manufacturer's specifications.

### Cell culture and reagents

The rat embryonic aortic smooth muscle cell line A7r5 (BCRC60082) was purchased from Bioresource Collection and Research Center (Taiwan), and cultured in Dulbecco's modified Eagle's medium (Gibco Laboratories, Grand Island, NY, USA) with 10% fetal bovine serum (Gibco Laboratories) and antibiotics. Cells were kept at 37°C in a humidified atmosphere with 5% CO_2_, and used at 80% confluence at passages 3–5 for the experiments. The medium was changed before experiments. The steroids E2 and resveratrol, an activator of *Sirt1* gene expression, were obtained from Sigma-Aldrich.

### Cell cycle analysis

We performed cell cycle analysis by analyzing DNA stained with propidium iodide by flow cytometry. We treated the A7r5 cells with drugs at the indicated concentrations, then harvested by trypsinization. We fixed the cells in 1 ml of 70% cold ethanol overnight, then stained the samples with staining buffer containing 0.01% propidium iodide, 0.1% sodium citrate, 0.3% Triton™ X-100, and 0.01% RNase A for 1 h in the dark. We determined the DNA content and cell cycle distribution using a BD FACSCalibur™ flow cytometer and CellQuest™ software (BD Biosciences, San Jose, CA, USA).

### Cell migration assay

We determined cell migration ability using a wound-healing assay. We plated the A7r5 cells in 6-well plates in medium without antibiotics, and treated the cells with drugs at the indicated concentrations. After 24 h, we wounded the cells with a sterile, plastic, 100-μl micropipette tip, then washed away the floating debris with PBS and cultured the cells in serum-free medium. We measured the width of the wound at different time points. We visualized and photographed 3 to 4 different locations under a phase-contrast inverted microscope.

### Cell viability assay

We determined cell viability using a conventional 3-(4,5-dimethylthiazol-2-yl)-2,5-diphenyltetrazolium bromide (MTT) assay. We dissolved the MTT in PBS at a concentration of 5 mg/ml. We added 20 μl MTT solution to each well, and incubated the plates at 37°C for 2 h. We terminated the assay by adding 100 μl of an aqueous solution of 20% SDS and 50% N,N,-dimethylsulfoxide to each well, then incubated the cells at 37°C for 1 h. Absorbance was measured with a plate reader at 570 nm to quantify the formazan, which reflects the number of viable cells in a culture.

### Statistical analysis

All experiments were repeated at least 3 times. The data are expressed as the mean ± standard error of the mean (SEM). Intergroup differences among 3 or more groups were analyzed using one-way analysis of variance. We used Student's *t*-test for comparison between 2 groups. Differences with a *p*-value < 0.05 were considered statistically significant.

## Abbreviations

SIRT1: sirtuin 1
VSMC: vascular smooth muscle cell
OVX: ovariectomized
E2: 17-β-estradiol
CVD: cardiovascular disease
IHC: immunohistochemical
PBS: phosphate-buffered saline
SDS: sodium dodecyl sulfate
TBST: buffer containing 20 mM Tris-HCl TWEEN^®^20
MTT: 3-(4,5-dimethylthiazol-2-yl)-2,5-diphenyltetrazolium bromide
SEM: standard error of the mean
Res: resveratrol
SD: standard deviation
